# IMPACT OF COMBINATIONS OF THE APOE Ɛ4 ALLELE AND TOMM40-APOC1 VARIANTS ON SURVIVAL TO OLDER AGES AND ALZHEIMER’S RISK

**DOI:** 10.1093/geroni/igac059.1732

**Published:** 2022-12-20

**Authors:** Alexander Kulminski, Ethan Jain-Washburn, Ian Philipp, Konstantin Arbeev, Eric Stallard, Mary Feitosa, Nicole Schupf, Kaare Christensen

**Affiliations:** Duke University, Durham, North Carolina, United States; DUKE UNIVERSITY, Durham, North Carolina, United States; Duke University, Durham, North Carolina, United States; Duke University, Durham, North Carolina, United States; Duke University, Durham, North Carolina, United States; Washington University, St Louis, Missouri, United States; Columbia University, New York, New York, United States; Southern Denmark University, Odense, Syddanmark, Denmark

## Abstract

Multifactorial diseases, health-related traits, and lifespan are polygenic phenotypes with complex genetic architectures. Polygenicity implies that multiple variants can impact the risks of these phenotypes independently or jointly. Recently, we showed that carriers of minor alleles of rs429358 (APOE ɛ4 encoding polymorphism), rs2075650 (TOMM40), and rs12721046 (APOC1) polymorphisms have up to 4.4 times higher risk of Alzheimer’s disease (AD) than APOE ɛ4 carriers without the minor alleles of rs2075650 and rs12721046. Here, we examined the chances of living to older ages—85 years and above—for carriers of compound genotypes combining these three polymorphisms using data from the Long Life Family Study, Framingham Heart Study, Cardiovascular Health Study, and UK Biobank. Consistent with their higher AD risk, carriers of the APOE ɛ4 allele with one or two minor alleles of rs2075650 and rs12721046 had 24.3% lower log odds of living to 85+ years (β=-0.243, p=2.22×10-2) than APOE ɛ4 carriers without either minor allele. Counterintuitively, AD did not mediate this risk. With AD-affected subjects excluded from the analysis, the effect size for the log odds of living to 85+ years (β=-0.352, p=2.35×10-3) was 1.45 times larger for APOE ɛ4 carriers with one or two minor alleles of rs2075650 and rs12721046. The chances of survival could be associated with lipid- and immunity-related mechanisms, whereas the risk of AD may be associated with amyloid-β-related mechanisms, among others. Targeting heterogeneous polygenic profiles of individuals at higher risks of multifactorial phenotypes may be a promising strategy for translating genetic discoveries to health care.

